# Supplementary material for: Effects of a Peptide Derived from the Primary Sequence of a Kallikrein Inhibitor Isolated from Bauhinia bauhinioides (pep-BbKI) in an Asthma–COPD Overlap (ACO) Model
Source: Int J Mol Sci. 2023 Jul 9;24(14):11261. doi: 10.3390/ijms241411261 (PMC10379932; doi:10.3390/ijms241411261)
Supplement: Supplementary file 1 [file ijms-24-11261-s001.zip › ijms-2455003-supplementary.pdf]

Supplementary Materials: The following

Table S1. Comparison of results of hyperresponsiveness to methacholine, cells of bronchoalveolar lavage fluid, inflammatory markers, remodeling markers, oxidative stress markers and signaling pathway, in airways and alveolar septa, between SAL and SAL-pep-BbKI control groups.

**Hyperresponsiveness to methacholine (%)**	**SAL**	**SAL-pep-BbKI**	**p ***
%Rrs	113.7±14.8	161.0±14.3	0.125
%Ers	77.3±7.5	52.7±8.7	0.110
%Raw	96.4±16.2	159.6±35.9	0.106
%Htis	39.0±5.6	25.1±2.8	0.137
%Gtis	54.4±9.9	85.2±13.6	0.091
**Bronchoalveolar lavage fluid (x10^{4}cells/mL)**	**SAL**	**SAL-pep-BbKI**	**p ***
Total cells	0,8±0,1	0.7±0.1	0.745
Eosinophils	0.2±0.05	0.1±0.03	0.221
Macrophages	0.2±0.03	0.1±0.03	0.343
Neutrophils	0.1±0.02	0.1±0.03	0.410
Lymphocytes	0.2±0.05	0.1±0.02	0.141
**Remodeling markers**	**SAL**	**SAL-pep-BbKI**	**p ***
MMP-9 - Airways (cells/10^{4}μm^2)	0.2±0.03	0.3±0.1	0.256

**Inflammatory marker (cells/10^{4}μm^2)**	**SAL**	**SAL-pep-BbKI**	**p ***
IL-1β - Airways	0.4±0.1	0.5±0.1	0.407
IL-1β - Alveolar septa	0.2±0.1	0.4±0.1	0.476
IL-4 - Airways	1.5±0.5	2.5±0.7	0.306
IL-4 - Alveolar septa	1.7±0.3	1.8±0.4	0.940
IL-5 - Airways	1.7±0.2	1.2±0.3	0.220
IL-5 - Alveolar septa	1.2±0.2	0.8±0.2	0.150
IL-6 - Airways	0.7±0.4	0.8±0.1	0.838
IL-6 - Alveolar septa	0.4±0.1	0.4±0.1	0.927
IL-10 - Airways	2.1±0.2	2.0±0.2	0.701
IL-10 - Alveolar septa	3.2±0.3	2.4±0.3	0.084
IL-13 - Airways	1.7±0.2	1.9±0.3	0.768
IL-13 - Alveolar septa	2.6±0.4	2.0±0.3	0.262
IL-17 - Airways	2.1±0.2	1.6±0.2	0.138

MMP-9 - Alveolar septa (cells/$10^4 \mu m^2$)	0.5±0.1	0.3±0.1	0.088	IL-17 - Alveolar septa	1.5±0.2	1.0±0.2	0.135
MMP-12 - Airways (cells/$10^4 \mu m^2$)	1.3±0.2	1.1±0.2	0.498	IFN-γ - Airways	0.7±0.2	0.4±0.1	0.125
MMP-12 - Alveolar septa (cells/$10^4 \mu m^2$)	0.4±0.1	0.5±0.1	0.728	IFN-γ - Alveolar septa	0.7±0.2	0.5±0.1	0.370
TGF-β - Airways (cells/$10^4 \mu m^2$)	0.8±0.3	0.8±0.1	0.854	TNF-α - Airways	1.7±0.4	3.2±0.9	0.077
TGF-β - Alveolar septa (cells/$10^4 \mu m^2$)	0.1±0.07	0.2±0.1	0.361	TNF-α - Alveolar septa	1.3±0.2	2.0±0.2	0.060
Collagen fibers - Airways (%)	1.6±0.4	2.1±0.5	0.553				
Collagen fibers - Alveolar septa (%)	2.4±0.2	2.7±0.2	0.432				
**Signaling pathway (cells/$10^4 \mu m^2$)**	**SAL**	**SAL-pep-BbKI**	**p ***	**Oxidative stress markers**	**SAL**	**SAL-pep-BbKI**	**p ***
NF-κB - Airways	0.4±0.1	1.0±0.2	0.343	iNOS - Airways (cells/$10^4 \mu m^2$)	3.3±0.5	2.1± 0.2	0.067
NF-κB - Alveolar septa	0.2±0.1	0.3±0.1	0.509	iNOS - Alveolar septa (cells/$10^4 \mu m^2$)	2.6±0.4	2.8 ±0.4	0.751
				Exhaled Nitric Oxide (ppb)	12.3±2.1	18.5±0.3	0.069

Note: Rrs, respiratory system resistance; Ers, respiratory system elastance; Raw, airway resistance; This, tissue elastance; Gtis, tissue resistance; IL, interleukin; IFN, interferon; TNF, tumor necrosis factor; MMP, metalloproteinase; TGF, transforming growth factor; iNOS, inducible nitric oxide synthase; NF, nuclear factor.
* p value.
